# Enhancing the shear strength of reinforced concrete deep beams using thin-diameter near-surface mounted steel wires: an experimental study

**DOI:** 10.1038/s41598-026-37355-8

**Published:** 2026-02-18

**Authors:** Mohamed Elkafrawy, Mohamed A. Altobgy, Sabry Fayed

**Affiliations:** 1 Structural Engineering Department, Faculty of Engineering, Tanta University, Tanta, Egypt; 2 Civil Engineering Department, Faculty of Engineering, Kafrelsheikh University, Kafrelsheikh, Egypt

**Keywords:** Shear, Deep beams, NSM, Wires, STM, Strengthening, Engineering, Materials science

## Abstract

This study investigates the effectiveness of near-surface mounted wire (NSMW) reinforcement as a shear-strengthening technique for reinforced concrete (RC) deep beams. Eleven specimens, including a control beam, were tested under three-point loading to evaluate the influence of vertical, horizontal, diagonal, and mesh NSMW configurations on shear behavior. The results showed that diagonal and mesh arrangements provided the greatest improvements in shear capacity, with increases of 61.8% and 59.1%, respectively, relative to the control. Vertical reinforcement also enhanced shear resistance by up to 50%, albeit with reduced deflection capacity, while horizontal reinforcement produced more modest gains, with a maximum increase of 34.1%. Among all schemes, the mesh configuration delivered the most balanced performance, achieving a 113.4% increase in energy absorption along with refined crack development. Crack pattern observations confirmed that diagonal and mesh layouts stabilized the compression strut, delayed diagonal cracking, and shifted failure away from the unreinforced shear span, whereas vertical and horizontal layouts were less effective in altering the failure mechanism. Overall, the findings highlight the novelty and practicality of thin-diameter NSMW reinforcement as a versatile alternative to conventional NSM bar techniques for strengthening RC deep beams.

## Introduction

 Enhancing the shear strength of RC beams has long been a critical challenge in structural engineering, driven by the need for safer and more resilient infrastructure. Conventional approaches, such as increasing concrete strength or providing transverse reinforcement (e.g., stirrups), are often insufficient under modern service loads and harsh environmental conditions. This has motivated the development of innovative strengthening methods, including optimized reinforcement layouts and advanced materials such as fiber-reinforced polymers (FRPs), ultra-high-performance concrete (UHPC), and engineered cementitious composites (ECC)^1,2^. Alongside material innovations, experimental and computational studies have improved understanding of shear behavior and enabled more reliable strengthening solutions^3,4^.

In parallel, sustainable approaches have gained momentum, with researchers investigating recycled concrete waste, electronic waste fibers, and green GFRP rebars to enhance structural performance^5–8^. Other studies have explored hybrid composites and wire mesh systems for improved confinement and capacity of structural members^9,10^. These advances highlight the growing focus on developing strengthening techniques that are both effective and practical.

Numerous experimental programs have evaluated shear strengthening strategies for RC members, reporting significant performance gains^11–13^. Externally bonded CFRP sheets, for example, have achieved shear strength increases of up to 138%^14,15^, while near-surface mounted (NSM) CFRP strips provide gains of 70–97% depending on configuration^16^. Alternative solutions include aluminum plates^17,18^, mechanical steel stitches^19^, Fe-based shape memory alloys (Fe-SMA)^20–22^, embedded steel or FRP bars^23^, and prestressed jackets^24^. UHPC-based retrofitting has also shown potential for altering failure modes and improving ductility^25^, while bolted side-plating has proven effective in fire-damaged members^26^.

Despite their success, these methods face important limitations. FRP systems are vulnerable to premature debonding and performance loss at elevated temperatures^27^. Textile-reinforced mortars (TRM) are limited by the low tensile capacity of cementitious matrices^28^, and strain-hardening cementitious composites (SHCCs), though ductile, do not fully restore stiffness and are underestimated in some design codes^29,30^. Aluminum retrofitting is less effective in low-strength concretes^21^, and UHPC solutions, while effective, remain costly and challenging to implement^31^.

Against this backdrop, near-surface mounted (NSM) reinforcement has emerged as a promising alternative. By embedding reinforcement in shallow grooves within the concrete cover and bonding with epoxy or mortar, NSM reinforcement offers advantages such as improved crack control, reduced debonding risk, and enhanced load–deflection behavior^32^. Compared to externally bonded systems, NSM reinforcement is better protected from environmental deterioration and typically produces more favorable failure modes dominated by reinforcement yielding followed by concrete crushing^33,34^.

The case of RC deep beams is particularly critical. Characterized by low span-to-depth ratios, deep beams are dominated by disturbed regions (D-regions), where conventional flexural theory does not apply and shear transfer is better described by strut-and-tie mechanisms^35,36^. Existing studies have demonstrated that NSM reinforcement can delay diagonal cracking, enhance strut stability, and significantly increase shear resistance in deep or heavily loaded members^37–39^. Nonetheless, most prior NSM research has concentrated on conventional steel bars or FRP laminates for flexural strengthening or in relatively shallow beam geometries.

To extend knowledge regarding the use of NSM in D-regions such as corbels, openings, dapped ends and deep beams, NSM wires is investigated in the current study. Use of thin-diameter (2.5 mm) near-surface mounted steel wires (NSMW) for shear strengthening of RC deep beams is studied. Thin wires offer several advantages compared with conventional NSM bars, including easier installation, smaller groove requirements, lower material cost, and greater flexibility in forming diverse reinforcement layouts. An experimental program of eleven RC deep beams was conducted, testing vertical, horizontal, diagonal, and mesh NSMW configurations under three-point bending. The study evaluates their influence on shear capacity, stiffness, crack development, and failure modes. By extending the NSM approach beyond its traditional applications, this work provides new insights into the effectiveness and practicality of NSMW systems as a cost-efficient and versatile solution for shear strengthening in RC deep beams.

## Aim of the study

The aim of this study is to investigate the effectiveness of using near-surface mounted (NSM) thin-diameter steel wires as a shear strengthening technique for RC deep beams. While NSM techniques using steel bars have been extensively studied—mainly in the context of flexural strengthening—the use of thin steel wires in enhancing shear behavior remains largely unexplored. Unlike conventional NSM bars (typically 10–12 mm in diameter), thin wires (2.5 mm) offer practical advantages such as easier installation, reduced groove dimensions, lower material costs, and greater flexibility in creating varied configurations. This research therefore examines the influence of different NSMW layouts (vertical, horizontal, diagonal, and mesh) and varying numbers of wires on the shear capacity, crack development, and failure modes of RC beams. By demonstrating that thin wires can achieve significant strength and ductility enhancements, the study underscores the novelty and practicality of this technique as a cost-effective alternative to traditional NSM bar applications.

## Experimental work

### Materials

The concrete beams tested in this study were cast using a consistent mix design. The mixture consisted of 1270 kg/m^3^ of graded crushed basalt dolomite (with a maximum aggregate size of 10 mm) as the coarse aggregate, 600 kg/m^3^ of locally sourced sand as the fine aggregate, 350 kg/m^3^ of Portland cement (42.5 grade), and 175 kg/m^3^ of water. Characteristics of these components are listed in Table 1. This mix achieved a characteristic compressive strength of 40 MPa after 28 days, in accordance with British Standards^40^.

**Fig. 1:**
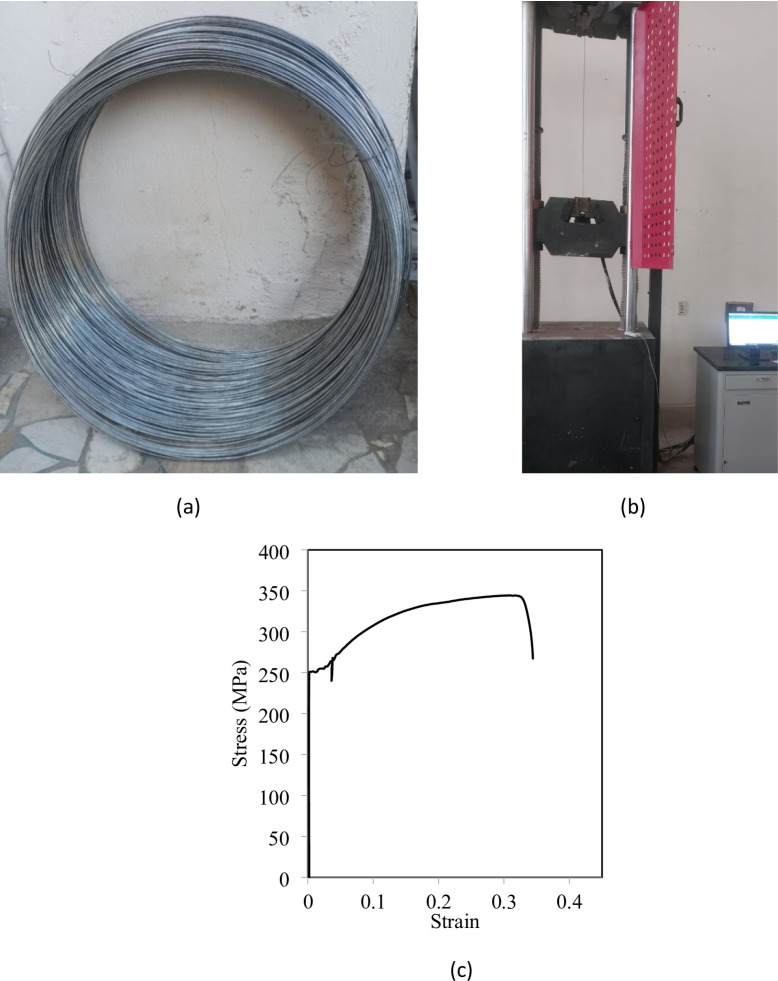
Characteristics of steel wires used. (**a**) Photo of steel wires used, (**b**) Set up of tension test, (**c**) Tensile stress-strain curve of wires.

For internal reinforcement, high-tensile steel (HTS) bars with a diameter of 16 mm (4Ø16) were used for both top and bottom longitudinal reinforcement to resist flexural stresses. Shear reinforcement was provided by 8 mm diameter normal mild steel (NMS) stirrups spaced at 50 mm intervals. The HTS bars had a yield strength of 448 MPa and an ultimate strength of 541 MPa, while the NMS stirrups had a yield strength of 253 MPa and an ultimate strength of 346 MPa. Table 2 shows characteristics of steel rebars used. The near-surface mounted wires used for external strengthening were smooth mild steel wires with a diameter of 2.5 mm, as illustrated in Fig. 1. Tension test was conducted on these wires to determine their properties, as illustrated in Fig. 1. Stress-strain curve of the wires is drawn in Fig. 1. These wires had a yield strength of 250 MPa, an ultimate strength of 350 MPa, a modulus of elasticity of 190 GPa, and an elongation capacity of 30%.

To anchor the wires within pre-cut grooves, a non-shrinking epoxy adhesive mortar, KEMAPOXY 165, was employed^41^. This two-component system consists of a modified epoxy resin and a compatible hardener, formulated to ensure strong bonding and long-term durability. Compliant with ASTM C881 specifications, KEMAPOXY 165 offers excellent mechanical properties and cohesive strength, making it particularly suitable for structural applications requiring reliable and high-performance bonding^42^. According to manufacturer, mechanical properties of this agent is listed in Table 3.

**Table 1:** Characteristics of concrete components.

Item	Size		Fineness modulus	Unit weight (kg/m^3^)	Specific gravity
	Diameter (mm)	Percentage (%)			
Crushed basalt	10	2.1	2	1200	2.4
River sand	2.36	3	4	1290	2.7
Cement	0.075	11	N/A	1220	3.15

**Table 2:** Characteristics of steel rebars used.

Item	Diameter (mm)	Use	Yield strength (MPa)	Ultimate strength (MPa)	Elasticity modulus (GPa)	Elongation (%)
HTS	16	Flexural reinforcement	448	541	200	11
NMS	8	Shear reinforcement	253	346	200	17
NSM wires	2.5	Strengthening	250	350	190	30

**Table 3:** Mechanical characteristics of the epoxy used.

Adhesive	Compression	Tension	Flexure	Bonding	Shear
KEMAPOXY 165	60–70 MPa	20–25 MPa	40–45 MPa	≈ 15 MPa	3-3.5 MPa

### Specimen details and setup

**Fig. 2:**
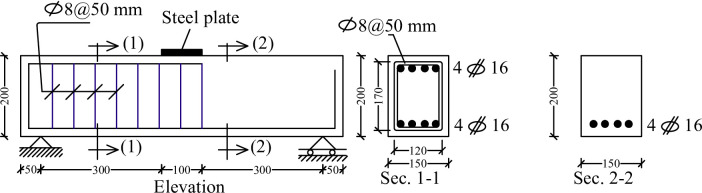
Details of the beams (mm).

A total of eleven RC deep beam specimens were cast and tested under a three-point loading configuration to assess the effectiveness of NSM thin steel wires in enhancing shear capacity. All specimens were designed with a span-to-depth ratio (a/d) of less than 2.0, thereby classifying them as deep beams in accordance with the provisions of ACI 318^43^. Each beam had an overall length of 800 mm, an effective span of 700 mm, a cross-sectional width of 150 mm, and a depth of 200 mm, with a clear concrete cover of 15 mm. For all samples in the practical program, the internal reinforcement was similar in both shear and bending. The beam sample was loaded with a single point in the middle. Divide the beam length into two equal halves; the left half is reinforced against shear while the right half was left without internal reinforcement in the shear until external strengthening using NSM could be added to that half. The internal longitudinal reinforcement consisted of four 16 mm HTS bars, arranged in the tension side along the beam span. For the left half of the beam, shear reinforcement was provided using 8 mm diameter mild steel stirrups at 50 mm spacing, as illustrated in Fig. 2, while no stirrups were used in the right half. To secure both the shear and the bending collapse in the left half of the beam, high flexural reinforcement and shear reinforcement density were used. Four 16 mm diameter bars were used on the top and bottom of the left half of the beam to increase its bending efficiency. This asymmetric reinforcement layout was deliberately chosen to induce shear failure in the opposite span (without stirrups), which also served as the zone for applying NSM wire strengthening. This configuration ensured that the shear response could be isolated and studied without interference from flexural failure. Section views 1–1 and 2–2 in Fig. 2 show the reinforcement details: Section 1–1 (stirrup-reinforced span) includes both longitudinal and transverse reinforcement, whereas Section 2–2 (shear-critical span) shows only longitudinal reinforcement, confirming the absence of stirrups.

**Fig. 3:**
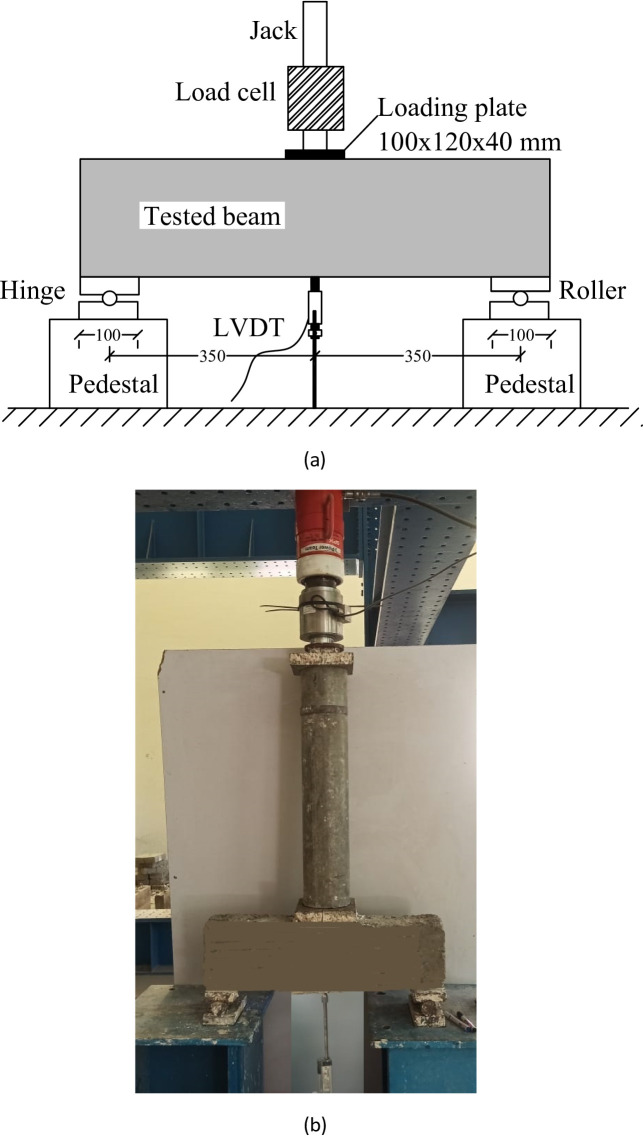
Testing set up. (**a**) Schematically diagram of test setup, (mm), (**b**) Real image of test setup.

The experimental setup is presented in Fig. 3. Each beam was simply supported, with a hinge at one end and a roller at the other to replicate realistic boundary conditions. A hydraulic jack applied a concentrated load at midspan through a steel loading plate (100 × 120 × 40 mm). The applied load was monitored using a calibrated 500 kN load cell positioned between the jack and the plate. Vertical deflections were recorded by a linear variable differential transformer (LVDT) located directly beneath the loading point. The clear span of 700 mm was divided into two equal shear spans of 350 mm each.

### Strengthening configuration

**Fig. 4:**
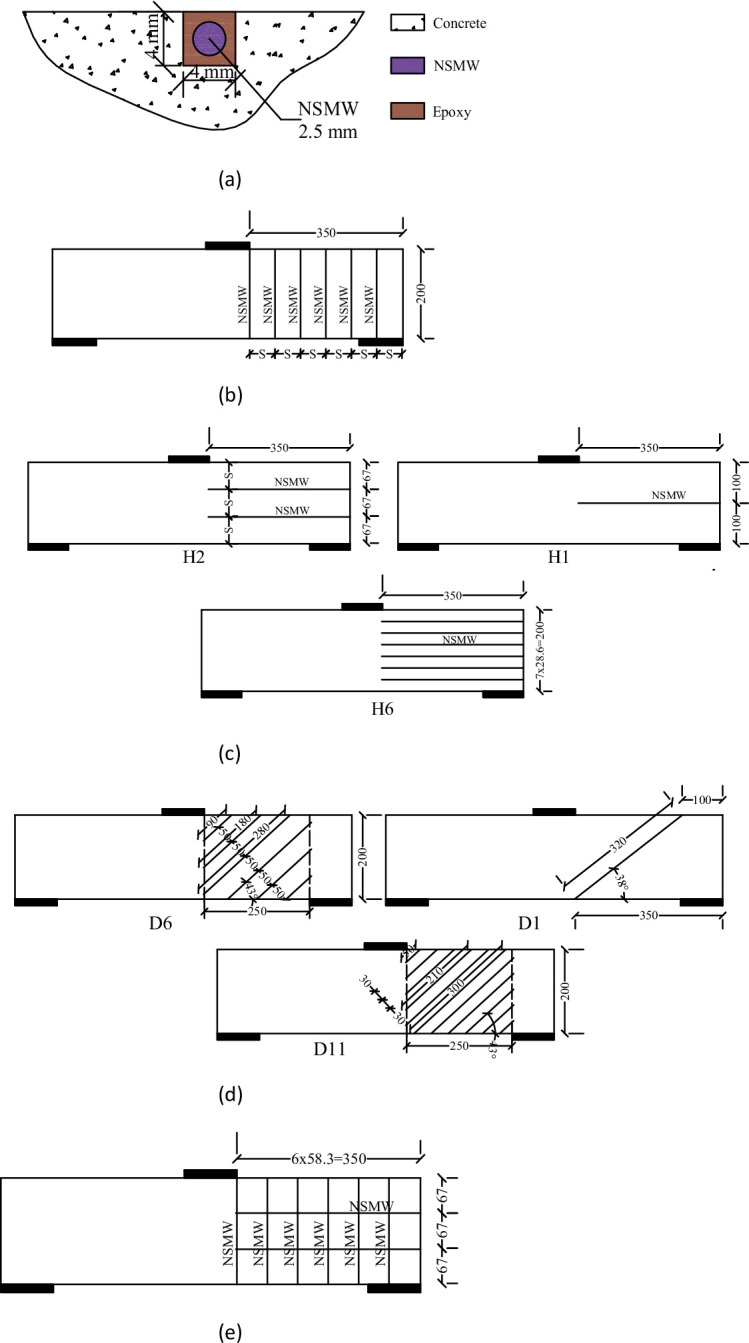
Strengthening of test beams. (**a**) Installation of NSMW, (**b**) Configuration of specimens vertically strengthened with NSMW, (V3, V6, V11), (**c**) Configuration of specimens horizontally strengthened with NSMW, (H1, H2, H6), (**d**) Configuration of specimens diagonally strengthened with NSMW, (D1, D6, D11), (**e**) Configuration of specimens horizontally and vertically strengthened with NSMW, (M6-2).

To examine the effectiveness of NSMW systems in shear strengthening, ten strengthened specimens and one unstrengthened control specimen were prepared, as summarized in Table 4. Strengthening was applied only to the shear-critical span (without stirrups) to ensure shear-dominant failure. The NSMWs, fabricated from 2.5 mm diameter mild steel, were installed in pre-cut grooves measuring 4 × 4 mm on the side faces of the beams. After placement, the grooves were filled with a high-strength, non-shrinking epoxy adhesive to ensure full bonding and effective stress transfer, as illustrated in Fig. 4a.

The strengthening schemes were categorized into four main configurations: vertical, horizontal, diagonal, and mesh. In the vertical scheme (specimens V3, V6, and V11), wires were placed perpendicular to the beam axis along the full height of the shear span, with 3, 6, and 11 wires corresponding to spacings of 117 mm, 58 mm, and 32 mm, respectively, and a constant embedment length of 200 mm (Fig. 4b). The horizontal scheme (H1, H2, H6) consisted of wires aligned parallel to the beam axis, where specimens were reinforced with 1, 2, or 6 wires spaced at 67 mm and 28.6 mm, each wire spanning 350 mm to cover the shear zone (Fig. 4c). In the diagonal scheme (D1, D6, D11), wires were inclined to intercept anticipated diagonal shear cracks, with D1 containing a single 320 mm wire, while D6 and D11 incorporated 6 and 11 wires spaced at 50 mm and 30 mm, respectively, with lengths ranging from 90 mm to 280 mm for adequate shear-span coverage (Fig. 4d). Finally, the mesh scheme (M6-2) combined six vertical wires (58.3 mm spacing, 200 mm length) and two horizontal wires (67 mm spacing, 350 mm length) to form a grid-like arrangement (Fig. 4e).

**Table 4:** Configuration of strengthening.

Beams	Scheme (group)	Spacing between wires S_w_ (mm)	No. of wires	Length of wires (mm)
C	–	-	–	–
V3	Vertical (GI)	117	3	200
V6		58	6	
V11		32	11	
H1	Horizontal (GII)	100	1	350
H2		67	2	
H6		28.6	6	
D1	Diagonal (GIII)	100	1	320
D6		50	6	90, 180, 280
D11		30	11	50–300
M 6-2	Mesh (GIV)	58.3 Vertical 67 Horizontal	6 Vertical NSMWs + 2 Horizontal NSMWs	200 mm for Vertical NSMWs and 350 mm for Horizontal NSMWs

## Results and discussion

### Cracks pattern and failure mode

**Fig. 5:**
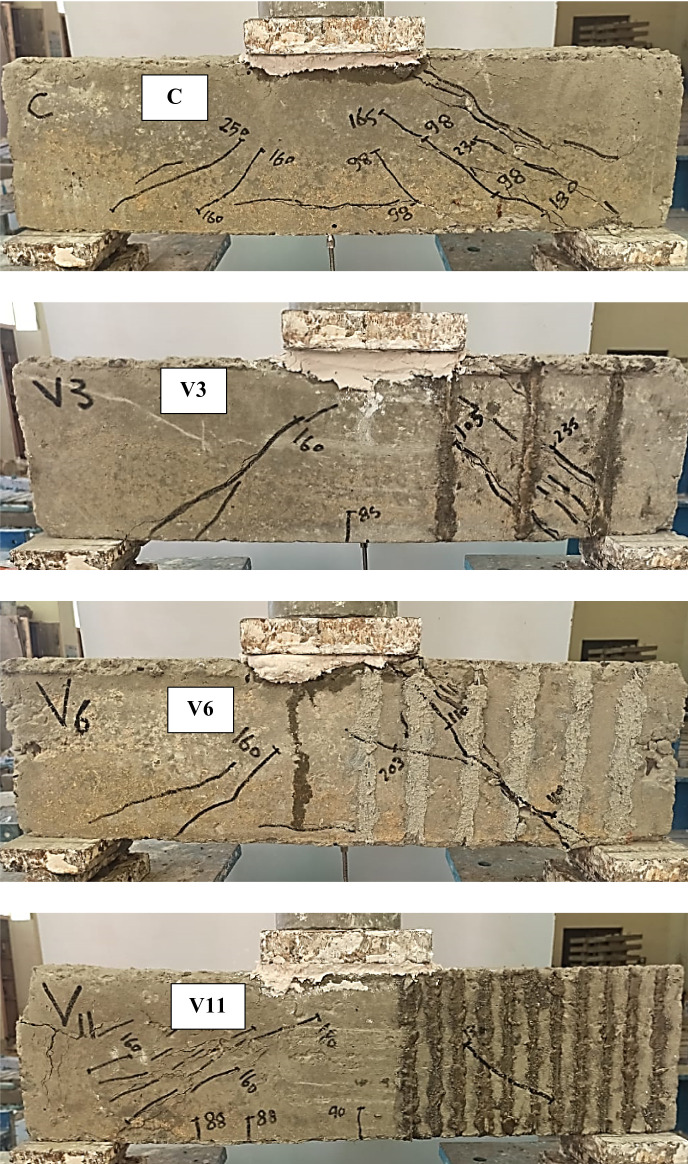

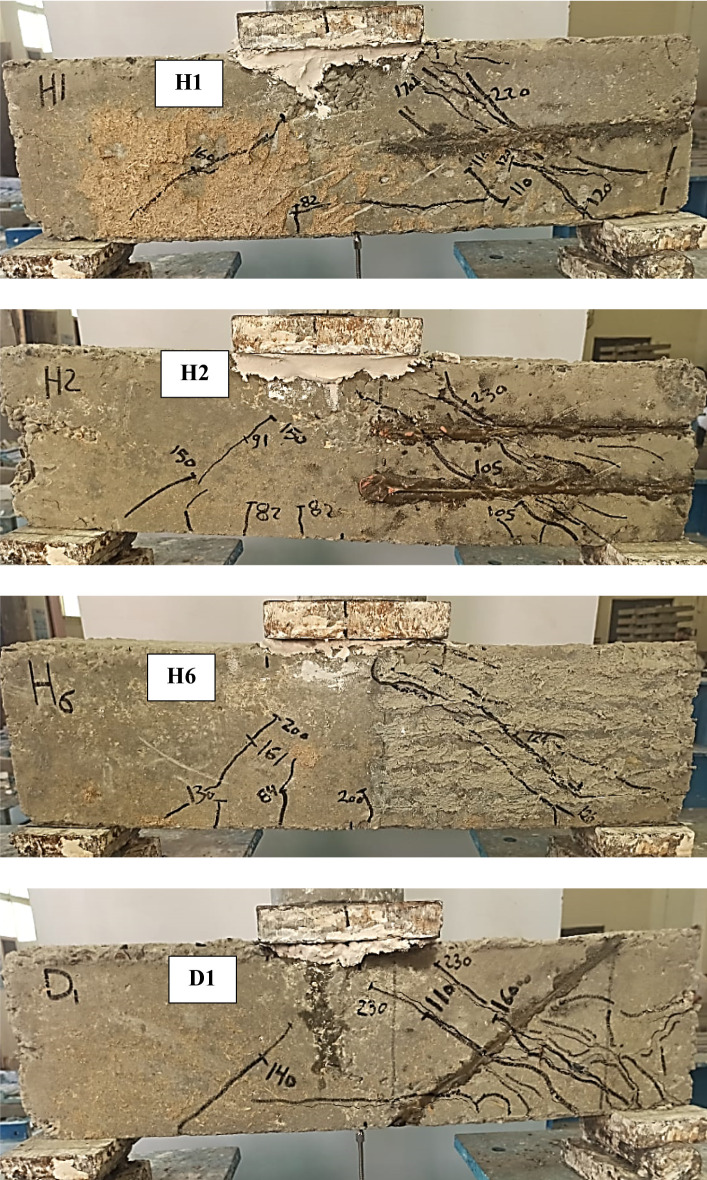

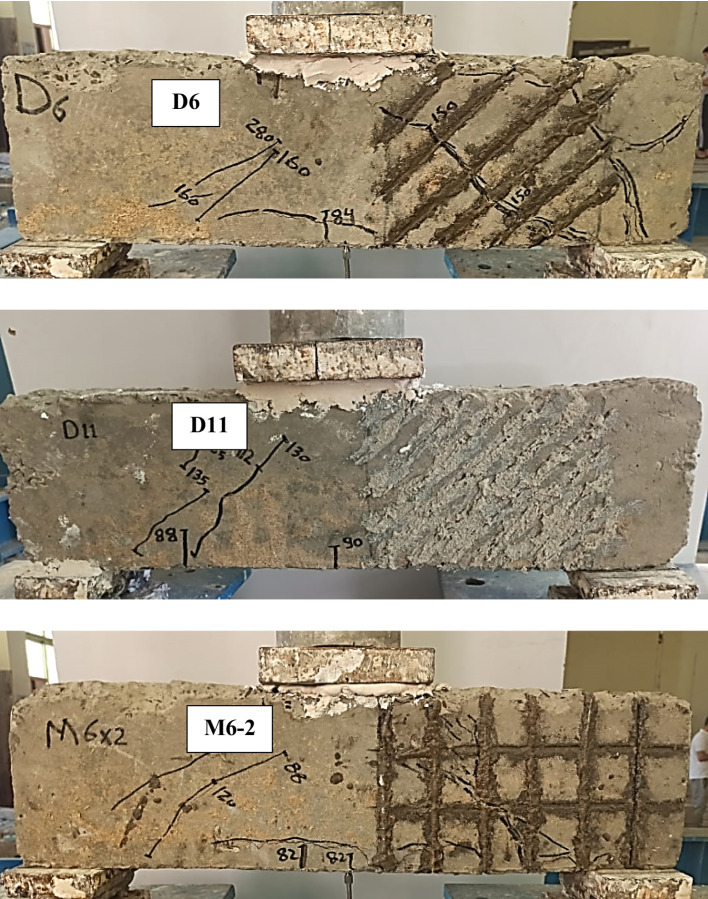
Cracks pattern of test beams.

The crack patterns and failure modes of the tested RC deep beams are presented in Fig. 5, and the corresponding mechanical performance is summarized in Table 5. The control specimen (C), which contained no transverse reinforcement or NSMW in the stirrup-free zone (SFZ), exhibited a brittle diagonal shear failure (SF) of the deep beams. A dominant diagonal strut developed between the loading point and the support, but once diagonal cracking formed along this strut, its load-carrying capacity was quickly compromised, leading to sudden failure. The first visible crack in the SFZ occurred at a load of 98 kN, while cracking in the stirrup zone (SZ) was delayed until 160 kN. The specimen ultimately failed in the SFZ at an ultimate load (Pu) of 220 kN, confirming the vulnerability of unreinforced shear spans in deep beams where strut integrity governs the overall shear resistance.

Beams strengthened with vertical NSMWs (V3, V6, V11) displayed improved crack control compared with the control beam. The additional vertical wires intersected diagonal cracks and helped delay the deterioration of the compression strut. The number of vertical wires directly influenced performance, with cracking becoming finer and failure modes shifting as reinforcement density increased. Specimen V11, with eleven vertical wires, achieved the best results in this group, showing a 32.6% increase in cracking load and a 50% increase in ultimate load compared to the control. Notably, V11 failed in the SZ rather than the SFZ, indicating that the dense vertical reinforcement effectively strengthened the strut in the critical span and redirected the shear demand to the untreated region. In contrast, V3 and V6 failed within the SFZ. Localized end debonding was observed in V6 where a major crack intersected a groove, but this did not significantly affect ultimate capacity as it occurred close to failure. All vertically strengthened beams exhibited reduced deflection at peak load, reflecting improved stiffness but a corresponding reduction in ductility.

Horizontally strengthened beams (H1, H2, H6) showed more horizontally oriented crack patterns, particularly in H6 with six wires. The horizontal wires, being aligned parallel to the main compression strut, offered limited resistance to its diagonal cracking and provided only modest improvements. Gains in shear capacity were moderate, with maximum increases of 28.6% in P_cr_ and 34.1% in P_u_. All failures occurred in the SFZ, underscoring the limited ability of horizontal reinforcement to confine or support the diagonal strut in deep beams. No debonding was observed, indicating reliable bonding performance. However, reductions in deflection at failure, along with relatively small gains in stiffness and energy absorption, confirm that horizontal reinforcement alone provides limited enhancement in shear performance.

The diagonal strengthening scheme (D1, D6, D11) was notably more effective. Diagonal wires aligned closely with the anticipated diagonal strut, allowing them to directly restrain its cracking and improve shear transfer. Cracks in these specimens were well distributed and finer compared to other groups, highlighting the ability of inclined reinforcement to intercept and stabilize the strut. Beam D11, with eleven diagonal wires, achieved the best overall structural response among all specimens, recording a 28.6% increase in Pcr, a 61.8% gain in Pu, and a remarkable 171.3% increase in absorbed energy (EA). Its failure occurred in the SZ, further confirming the effectiveness of the strengthening in shifting the failure zone away from the treated span. Minor debonding was observed in D6 near ultimate load, but it had little effect on performance. Overall, diagonal strengthening provided a favorable balance between stiffness and ductility by supporting the strut action and enhancing energy absorption capacity.

The mesh-strengthened beam (M6-2), which combined vertical and horizontal wires, exhibited the most refined crack distribution and outstanding overall performance. In this case, the hybrid grid arrangement provided multidirectional restraint that confined and stabilized the diagonal strut, thereby improving both shear strength and ductility. Cracking was well distributed in multiple directions, effectively controlling both vertical and diagonal shear cracks. M6-2 recorded the highest cracking load of 160 kN (63.3% increase over the control) and an ultimate load of 350 kN (59.1% increase). Although ultimate deflection decreased by 30.8%, stiffness improved by 138.7%, and absorbed energy more than doubled, with a 113.4% gain. This demonstrates the superior ability of the hybrid mesh arrangement to optimize both strength and energy dissipation in shear-critical deep beams.

**Table 5:** Summary of test results.

Beam ID	Failure of strengthened zone	*P*_cr_ (kN)	Gain in Pcr (%)	*P*_u_ (kN)	Gain in Pu (%)	δ_u_ (mm)	Gain in δ_u_ (%)	k (kN/mm)	Gain in k (%)	EA (kN.mm)	Gain in EA (%)
C	SF	98	–	220	–	4.77	–	80	–	790	–
V3	SF + wire slip	105	7.1	238	8.2	4.18	− 12.4	94	17.5	1089	37.8
V6	SF + wire slip	110	12.2	270	22.7	3.47	− 27.2	110	37.5	1317	66.7
V11	No	130	32.6	330	50.0	3.30	− 30.8	148	85.0	1449	83.4
H1	SF	110	12.2	220	0.0	4.76	− 0.2	80	0.4	792	0.2
H2	SF	105	7.1	260	18.2	3.47	− 27.2	130	62.5	923	16.8
H6	SF	126	28.6	295	34.1	3.70	− 22.4	92	15.0	941	19.1
D1	SF	110	12.2	260	18.2	4.52	− 5.2	81	1.2	1665	110.8
D6	SF	115	17.3	287	30.4	4.50	− 5.7	86	7.5	1731	119.1
D11	No	126	28.6	356	61.8	4.34	− 9.0	100	25.0	2143	171.3
M6-2	No	160	63.3	350	59.1	3.30	− 30.8	191	138.7	1686	113.4

### Load-deflection curves

**Fig. 6:**
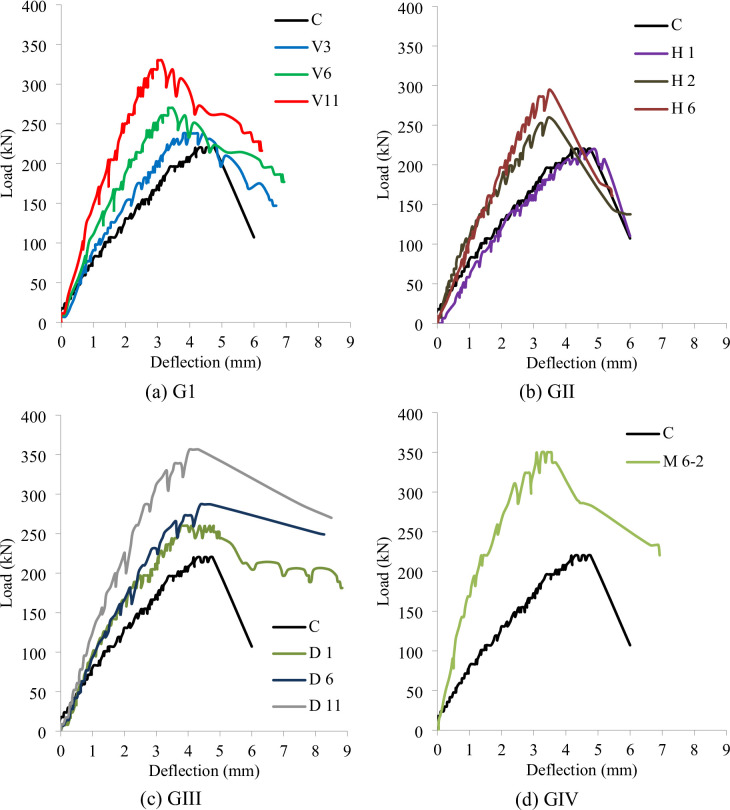
Load-deflection curves of test beams. (**a**) G1, (**b**) GII, (**c**) GIII, (**d**) GIV.

The load–deflection responses of the tested RC deep beams under three-point bending are presented in Fig. 6, grouped by strengthening configuration. These curves highlight the influence of NSMW reinforcement on stiffness, ductility, and ultimate load capacity, with the control beam (C) serving as the baseline. For deep beams, where load transfer is dominated by diagonal strut-and-tie action, the curves provide insight into how different strengthening layouts influenced the integrity and stability of the compression strut and the overall shear resistance.

In Group G1 (Fig. 6a), beams V3, V6, and V11, strengthened with vertical NSMWs, exhibited a pronounced increase in load capacity compared to the control. The ascending branch of the curve for V11 was notably steeper, reflecting the higher initial stiffness provided by dense vertical reinforcement intersecting the diagonal strut. V11 reached the highest ultimate load but experienced a sharper post-peak decline, indicating reduced ductility as the strengthened strut failed abruptly. V3 and V6 showed moderate strength gains with reduced deflections at failure. The shortened post-peak branches across this group suggest that while vertical reinforcement enhanced strut capacity, it also promoted a shift toward more brittle behavior typical of shear-dominated deep beams.

Group G2 (Fig. 6b) presents horizontally strengthened specimens H1, H2, and H6. Their curves were generally closer to the control beam, especially H1, which displayed negligible improvement. H2 and H6 achieved slightly higher stiffness and ultimate loads, as reflected in steeper ascending branches, but failure occurred with deflections comparable to the control. Since horizontal wires were aligned parallel to the main diagonal strut, they contributed little to restraining its cracking, limiting their effectiveness. The overall response remained relatively ductile, with modest stiffness gains but without a significant shift in the dominant shear failure mode.

The curves in Group G3 (Fig. 6c), corresponding to diagonal strengthening schemes (D1, D6, D11), demonstrated the most effective improvements. These specimens showed substantially steeper ascending branches and higher ultimate loads, confirming the direct contribution of diagonal NSMWs in stabilizing the compression strut and bridging diagonal cracks. Beam D11, in particular, exhibited superior performance, combining high load-bearing capacity with a smoother post-peak decline, suggesting a more gradual and controlled failure. The larger areas under the D6 and D11 curves indicate enhanced energy absorption, reflecting a favorable balance between stiffness and ductility. This highlights the effectiveness of aligning reinforcement with the natural force flow path in deep beams.

Group G4 (Fig. 6d) compares the mesh-strengthened beam (M6-2) with the control. M6-2 displayed the steepest initial slope, confirming the highest stiffness, and achieved a markedly higher peak load. Although ultimate deflection was reduced, the beam sustained loading over a wider range, with a broad area under the curve indicating excellent energy dissipation. The multidirectional mesh configuration confined and stabilized the diagonal strut more effectively than single-orientation schemes, resulting in refined crack distribution and superior structural integrity. Overall, the load–deflection response of M6-2 reflects the most balanced behavior, combining high strength, stiffness, and energy absorption, while effectively enhancing the shear performance of deep beams.

### Ultimate load and corresponding Deflection

**Fig. 7:**
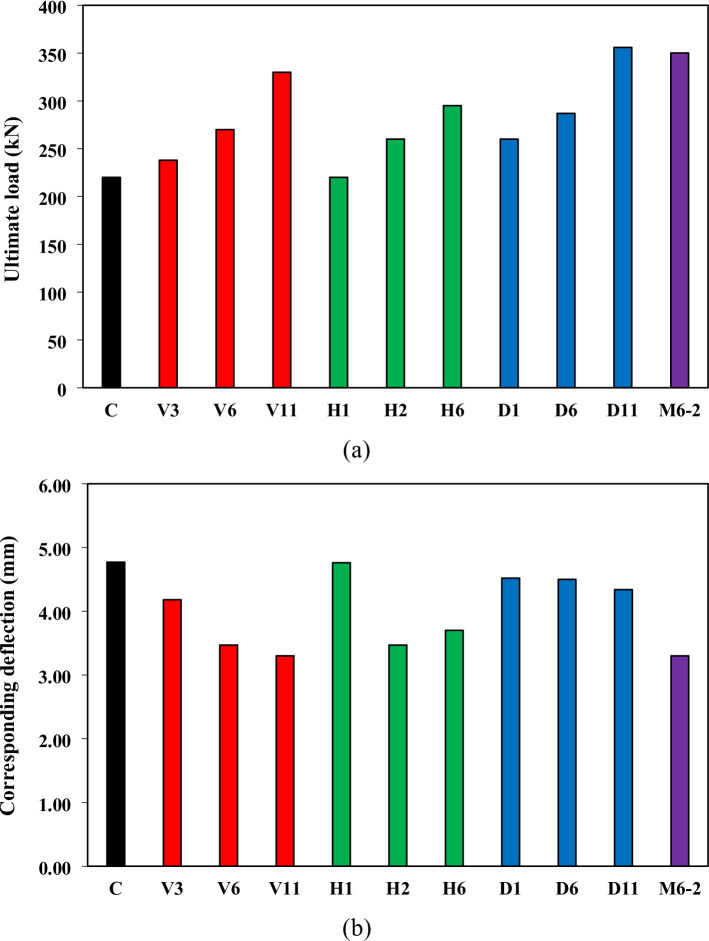
(**a**) Ultimate load and (**b**) corresponding deflection comparison.

Figure 7 compares the ultimate load (P_u_) and corresponding deflection (δ_u_) of all tested RC deep beams. The control beam (C) reached a P_u_ of 220 kN, serving as the baseline. All beams strengthened with NSMW reinforcement achieved higher ultimate loads, with gains ranging from 8.2% in V3 to 61.8% in D11, confirming the positive effect of NSMWs in enhancing shear capacity. The diagonal (D11) and mesh (M6-2) configurations were the most effective, reaching P_u_ values of 356 kN and 350 kN, respectively, followed closely by dense vertical reinforcement (V11), which achieved a 50% increase. These findings emphasize that reinforcement layouts aligned with or confining the main diagonal strut of the deep beam provide the greatest improvements in shear resistance.

The corresponding deflections at ultimate load are shown in Fig. 7b. Most strengthened specimens exhibited reduced δ_u_ relative to the control, reflecting increased stiffness and restrained deformation. The reductions were most pronounced in V11 and M6-2 (both 30.8%) and V6 (27.3%). Only H1, with a single horizontal wire, showed negligible change (− 0.2%). While reduced deflections are consistent with improved stiffness, they also point to a reduction in ductility, highlighting a key trade-off in strengthening deep beams: enhancing strut stability and strength often comes at the expense of deformation capacity.

### Stiffness

**Fig. 8:**
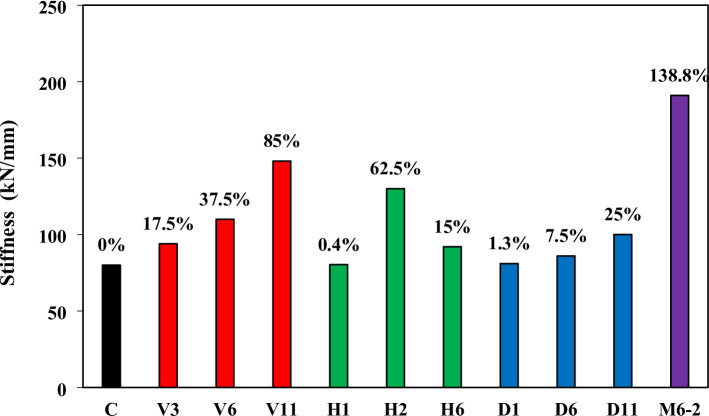
Stiffness comparison.

Stiffness, a key indicator of structural rigidity, was evaluated from the slope of the initial segment of the load–deflection (P–δ) curve. Figure 8 compares the stiffness of the tested beams, with the control (C) establishing a baseline of 80 kN/mm. The mesh configuration (M6-2) achieved the highest improvement, with a 138.8% gain, demonstrating the efficiency of multidirectional reinforcement in confining the diagonal strut and resisting deformation. Vertical strengthening also provided significant benefits, with V11, V6, and V3 recording gains of 85%, 37.5%, and 17.5%, respectively, confirming the positive correlation between reinforcement density and stiffness enhancement. Horizontal reinforcement showed mixed results: H2 achieved a 62.5% increase, while H6 and H1 recorded 15% and 0.4%, respectively, indicating that only multiple horizontal wires contribute meaningfully to rigidity. Diagonal reinforcement delivered modest stiffness gains, with increases of 25% (D11), 7.5% (D6), and 1.3% (D1). Overall, the results show that stiffness improvement in deep beams is most effective when reinforcement either directly intersects the strut (dense verticals) or confines it from multiple directions (mesh), while horizontal or limited diagonal layouts provide less influence on initial rigidity.

### Energy absorption capacity

**Fig. 9:**
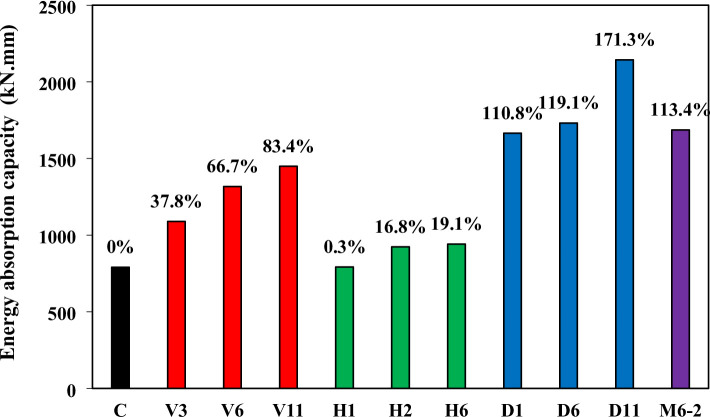
Energy absorption capacity comparison.

Energy absorption capacity (EAC), defined as the total area under the load–deflection curve^42^, reflects a beam’s ability to dissipate energy under shear-dominated loading and is closely associated with ductility. Figure 9 presents the EAC results. The control beam (C) exhibited a baseline of 790 kN.mm. Diagonal reinforcement delivered the largest improvements, with D11 achieving a remarkable 171.3% increase, followed by D6 (119.1%) and D1 (110.8%). These results highlight the strong capacity of diagonally oriented wires to stabilize the strut, delay diagonal cracking, and enable higher energy dissipation. Vertical reinforcement also performed well, with V11, V6, and V3 showing increases of 83.4%, 66.7%, and 37.8%, respectively. In contrast, horizontal reinforcement contributed only modestly, with gains of 16.8% (H2), 19.1% (H6), and virtually none in H1 (0.3%). The mesh configuration (M6-2) combined the advantages of vertical and horizontal reinforcement, producing a 113.4% increase in EAC, which demonstrates its balanced capacity to enhance both strength and ductility. Collectively, these results show that reinforcement schemes aligned with or confining the strut (diagonal and mesh) are most effective in improving the resilience of deep beams, while horizontal layouts are least effective.

## Conclusion

This study investigated the effectiveness of NSMW reinforcement as a shear strengthening technique for reinforced concrete deep beams. Eleven beams, including a control specimen, were tested under three-point loading to evaluate the impact of vertical, horizontal, diagonal, and mesh reinforcement schemes. The results were analyzed in terms of shear capacity, stiffness, crack control, and energy absorption. The main conclusions are as follows:

- Crack patterns and failure modes varied with the strengthening configuration. The control beam exhibited brittle diagonal shear failure, while NSMW-strengthened beams showed more refined and distributed cracking. Diagonal and mesh layouts were most effective in stabilizing the compression strut and shifting the failure away from the stirrup-free zone, confirming their role in delaying shear crack propagation and enhancing overall structural integrity.
- Diagonal NSMW reinforcement was the most effective scheme, with specimen D11 achieving a 61.8% increase in shear capacity relative to the control. This configuration efficiently intercepted diagonal shear cracks, stabilized the concrete strut, and delivered the highest improvement in energy absorption (171.3%).
- Mesh reinforcement (M6-2), combining vertical and horizontal wires, provided a balanced structural response. It achieved a 59.1% gain in shear capacity, a 113.4% increase in energy absorption, and notable improvements in stiffness, making it an effective option where both strength and ductility are required.
- Vertical reinforcement significantly improved shear resistance, with V11 achieving a 50% increase. However, this came with reduced deflection capacity due to increased stiffness, reflecting a strength–ductility trade-off.
- Horizontal reinforcement was the least effective, with H6 showing only a 34.1% increase in shear capacity. While some crack control benefits were observed, horizontal NSMW did not significantly improve strut performance compared with diagonal and mesh layouts.

## Data Availability

The datasets used and/or analyzed during the current study available from the corresponding author on reasonable request.
